# Interoperable, Domain-Specific Extensions for the German Corona Consensus (GECCO) COVID-19 Research Data Set Using an Interdisciplinary, Consensus-Based Workflow: Data Set Development Study

**DOI:** 10.2196/45496

**Published:** 2023-07-18

**Authors:** Gregor Lichtner, Thomas Haese, Sally Brose, Larissa Röhrig, Liudmila Lysyakova, Stefanie Rudolph, Maria Uebe, Julian Sass, Alexander Bartschke, David Hillus, Florian Kurth, Leif Erik Sander, Falk Eckart, Nicole Toepfner, Reinhard Berner, Anna Frey, Marcus Dörr, Jörg Janne Vehreschild, Christof von Kalle, Sylvia Thun

**Affiliations:** 1 Core Facility Digital Medicine and Interoperability, Berlin Institute of Health at Charité – Universitätsmedizin Berlin, Berlin, Germany; 2 Institute of Medical Informatics, Charité – Universitätsmedizin Berlin, Corporate Member of Freie Universität Berlin and Humboldt-Universität zu Berlin, Berlin, Germany; 3 Department of Anesthesia, Critical Care, Emergency and Pain Medicine, Universitätsmedizin Greifswald, Greifswald, Germany; 4 Department Interoperability, Digitalization and IT, National Association of Statutory Health Insurance Physicians, Berlin, Germany; 5 Joint Charité and BIH Clinical Study Center, Berlin Institute of Health at Charité – Universitätsmedizin Berlin, Berlin, Germany; 6 Charité - Universitätsmedizin Berlin, Corporate Member of Freie Universität Berlin and Humboldt-Universität zu Berlin, Berlin, Germany; 7 Department of Infectious Diseases and Respiratory Medicine, Charité – Universitätsmedizin Berlin, Corporate Member of Freie Universität Berlin and Humboldt-Universität zu Berlin, Berlin, Germany; 8 Department of Tropical Medicine, Bernhard Nocht Institute for Tropical Medicine, Hamburg, Germany; 9 Department of Medicine I, University Medical Centre Hamburg-Eppendorf, Hamburg, Germany; 10 Department of Pediatrics, University Hospital Carl Gustav Carus, Technische Universität Dresden, Dresden, Germany; 11 Medical Clinic and Policlinic I, University Hospital of Würzburg, Würzburg, Germany; 12 Department of Internal Medicine B, Universitätsmedizin Greifswald, Greifswald, Germany; 13 Partner Site Bonn-Cologne, German Centre for Infection Research, Cologne, Germany; 14 Department I of Internal Medicine, University Hospital of Cologne, Cologne, Germany; 15 Department II of Internal Medicine, Hematology/Oncology, Goethe University, Frankfurt am Main, Germany

**Keywords:** interoperability, research data set, Fast Healthcare Interoperability Resources, FHIR, FAIR principle, COVID-19, interoperable, SARS-CoV-2, pediatric, immunization, cardiology, standard

## Abstract

**Background:**

The COVID-19 pandemic has spurred large-scale, interinstitutional research efforts. To enable these efforts, researchers must agree on data set definitions that not only cover all elements relevant to the respective medical specialty but also are syntactically and semantically interoperable. Therefore, the German Corona Consensus (GECCO) data set was developed as a harmonized, interoperable collection of the most relevant data elements for COVID-19–related patient research. As the GECCO data set is a compact core data set comprising data across all medical fields, the focused research within particular medical domains demands the definition of extension modules that include data elements that are the most relevant to the research performed in those individual medical specialties.

**Objective:**

We aimed to (1) specify a workflow for the development of interoperable data set definitions that involves close collaboration between medical experts and information scientists and (2) apply the workflow to develop data set definitions that include data elements that are the most relevant to COVID-19–related patient research regarding immunization, pediatrics, and cardiology.

**Methods:**

We developed a workflow to create data set definitions that were (1) content-wise as relevant as possible to a specific field of study and (2) universally usable across computer systems, institutions, and countries (ie, interoperable). We then gathered medical experts from 3 specialties—infectious diseases (with a focus on immunization), pediatrics, and cardiology—to select data elements that were the most relevant to COVID-19–related patient research in the respective specialty. We mapped the data elements to international standardized vocabularies and created data exchange specifications, using Health Level Seven International (HL7) Fast Healthcare Interoperability Resources (FHIR). All steps were performed in close interdisciplinary collaboration with medical domain experts and medical information specialists. Profiles and vocabulary mappings were syntactically and semantically validated in a 2-stage process.

**Results:**

We created GECCO extension modules for the immunization, pediatrics, and cardiology domains according to pandemic-related requests. The data elements included in each module were selected, according to the developed consensus-based workflow, by medical experts from these specialties to ensure that the contents aligned with their research needs. We defined data set specifications for 48 immunization, 150 pediatrics, and 52 cardiology data elements that complement the GECCO core data set. We created and published implementation guides, example implementations, and data set annotations for each extension module.

**Conclusions:**

The GECCO extension modules, which contain data elements that are the most relevant to COVID-19–related patient research on infectious diseases (with a focus on immunization), pediatrics, and cardiology, were defined in an interdisciplinary, iterative, consensus-based workflow that may serve as a blueprint for developing further data set definitions. The GECCO extension modules provide standardized and harmonized definitions of specialty-related data sets that can help enable interinstitutional and cross-country COVID-19 research in these specialties.

## Introduction

The COVID-19 pandemic has led to unprecedented, strong efforts in connecting nationwide and international research to help manage the disease and its effects on public health. To enable research across different health care providers, institutions, or even countries, interoperability between medical data systems is essential [1]. Therefore, early in the pandemic, the German Corona Consensus (GECCO) data set was developed in a collaborative effort to provide a standardized, unified core data set for interinstitutional COVID-19–related patient research [2]. The GECCO data set specifies a set of 81 essential clinical data elements from 13 domains, such as anamnesis and risk factors, symptoms, and vital signs, that have been selected by expert committees from university hospitals, professional associations, and research initiatives. Since its development, the GECCO data set has been implemented in a large number of institutions, most notably in virtually all German university hospitals, which now provide access to the GECCO data set in the context of the German COVID-19 Research Network of University Medicine (“Netzwerk Universitätsmedizin”) [3,4].

The GECCO data set was developed to contain as many relevant data elements as possible but few enough to keep the effort of implementing the data set manageable. Therefore, the data set contains mostly data elements of general research interest, excluding data elements that are only of interest for particular medical specialties or use cases. These data items are considered part of domain-specific extension modules of the GECCO data set, which are introduced in this paper.

We aimed to develop domain-specific extensions to the GECCO data set that cover the most relevant data elements for COVID-19–related patient research in the infectious disease (with a focus on immunization), pediatrics, and cardiology medical specialties. To that end, we first developed a workflow that aims at providing data set definitions that (1) contain the most relevant data elements for the research aims of the end users and (2) can be applied universally across institutions and countries. We then followed that workflow with different groups of medical experts from different medical specialties to define extension modules that are relevant for research regarding immunization, pediatrics, and cardiology.

These extension modules complement the GECCO core data set and use the same international health IT standards and terminologies as those in the GECCO data set, such as the Systematized Nomenclature of Medicine-Clinical Terms (SNOMED CT) [5], the Logical Observation Identifiers Names and Codes (LOINC) [6,7], and the Fast Healthcare Interoperability Resources (FHIR) [8,9] standard. The extension modules were developed in close alignment with the GECCO data set to ensure interoperability and compatibility with existing definitions.

We herein describe the consensus-based data element selection and data format definition workflow that we applied in close collaboration with medical experts from 3 specialties—infectious diseases (with a focus on immunization), pediatrics, and cardiology (ie, for content definition)—as well as medical information specialists and FHIR developers (ie, for technical aspects). This workflow may serve as a blueprint for the further development of consensus-based data set definitions.

## Methods

### Workflow Definition

We aimed to develop a workflow to create data set definitions that are (1) content-wise as relevant as possible to a specific field of study and (2) universally usable across computer systems, institutions, and countries (ie, interoperable). We based the specification of the workflow on our experience with the definition of the GECCO data set, during which health professionals from 50 institutions (university hospitals, professional associations, and other relevant organizations) participated to define the most relevant data elements for general-scope, COVID-19–related research [2]. To fulfill the first requirement (relevancy), we decided to leave the full responsibility of data element selection to groups of medical professionals of the respective specialty, with only minimal interference by the medical information specialists. We deliberately did not specify the exact process of how the group of medical experts could select the data elements (eg, literature review, focus groups, and consensus-based processes) to allow for the maximal flexibility of the data set definition workflow, with respect to the medical experts’ values and preferences. To fulfill the second requirement (interoperability), we adopted a model that was loosely based on the data FAIRification workflow of Jacobsen et al [10]; the mapping, quality assurance, and publication steps are outlined in detail below.

### Selection of Data Items

**Figure 1.**
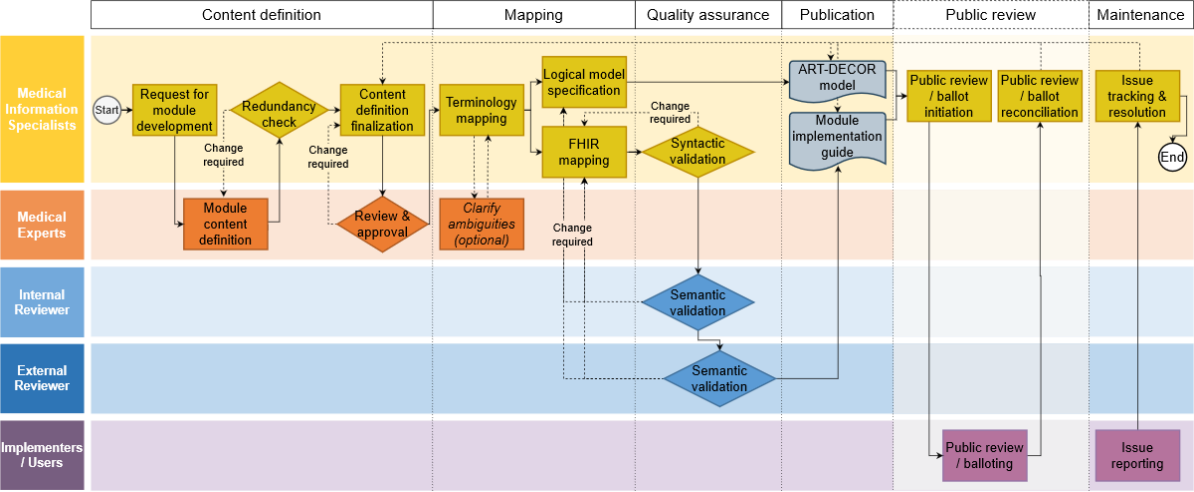
Flowchart of the consensus-based, interdisciplinary data set definition and mapping workflow for the domain-specific COVID-19 research data sets. FHIR: Fast Healthcare Interoperability Resources.

The content of the domain-specific research data sets was defined by medical domain experts in a transparent workflow (Figure 1). The involvement of the medical domain experts as the end users of the data to be provided ensured that the contents of the data sets were aligned to the actual research needs. In our project, the so-called *subject- and organ-specific working groups* of the National Pandemic Cohort Network (“Nationales Pandemie Kohorten Netz” [NAPKON]) served as the domain-specific groups of medical experts. These groups were established by a voluntary association of medical experts from the medical specialties within the nationwide NAPKON project in Germany. Each of the subject- and organ-specific working groups elected a board, and all communication between the data set developers and the working groups was organized and carried out via the working groups’ boards. In preparation for the GECCO extension modules, we invited the subject- and organ-specific groups for infectious diseases (with a focus on immunization), pediatrics, and cardiology to provide up to 50 data elements (with up to 10 response items each) that were, in the view of the medical experts, the most relevant to patient-related COVID-19 research in these medical specialties and not already included in the GECCO core data set. If necessary, more data items or response options could be provided in coordination with the medical information specialists. The provided data items were then reviewed by the medical information specialists, and a first definition of the contents of the extension module was returned to the respective subject- and organ-specific working group for approval or change requests. After approval by the subject- and organ-specific working group, the definition of the extension module content was considered finalized.

### Development of the Standardized Data Formats

To map the data items selected by the subject- and organ-specific working groups to international standard vocabularies, we performed a consensus-based mapping procedure, wherein every concept was mapped to appropriate vocabularies—the SNOMED CT for general concepts [11]; LOINC for observations [7]; *International Statistical Classification of Diseases and Related Health Problems, 10th Revision, German Modification* for diagnoses [12]; Anatomical Therapeutic Chemical Classification System for Germany for drugs and active ingredients [13]; and Unified Code for Units of Measure for measurement units [14]—by 2 medical information specialists independently. Ambiguities and nonmatching mappings were then discussed among the medical information specialists and in close collaboration with the medical experts of the subject- and organ-specific working groups until consensus was achieved. The data item–to-concept mappings were annotated on ART-DECOR, an open-source collaboration platform for creating and maintaining data set element descriptions [15].

As for the GECCO data set, the format for data exchange was specified by using Health Level Seven International (HL7) FHIR resources. The mapping of data items to FHIR resources was performed in an iterative, consensus-based workflow among the medical information specialists. Wherever possible, published FHIR profiles from the GECCO data set, the Medical Informatics Initiative [16], or the National Association of Statutory Health Insurance Physicians (“Kassenärztliche Bundesvereinigung”) [17]—in this order of priority—served as the base definition for the future extension module profiles.

The profiles and value sets were specified by using the FHIR Shorthand (FSH) language (version 1.2.0) and translated to Structure Definition JSON files by using the HL7 FSH SUSHI software package (version 2.2.3) [18,19]. We required that at least one exemplary instance be defined for every profile. The syntactic validation of the profile and value set definitions was performed through the error-free conversion of the FSH files to JSON via SUSHI, and the subsequent validation of each profile and their defined instances was performed by using the HL7 FHIR validator as implemented in the FSH Validator Python package (version 0.2.2) [20]. After the successful syntactic validation of a set of profiles, the profiles were subjected to a 2-stage review process, as follows. First, the profiles and the corresponding value sets and extensions were internally reviewed for semantic appropriateness with the GECCO core developer (JS). After all necessary changes and approval by the internal reviewer, the profiles were subjected to the second review round by an external FHIR development expert. Subsequent to necessary corrections and the approval of the external reviewer, the respective profiles, together with their value sets and, optionally, extensions and code systems, were considered finalized and published to the main branch of the Git repository. The subsequent and ongoing maintenance phase of the data set definitions involves inviting implementers and users to report any issues that they encounter with the definitions, in order to ensure their accuracy and relevance over time.

The whole development process was performed collaboratively on GitHub. The syntactic validation of the profiles was performed via continuous integration/continuous development workflows, which were implemented as GitHub actions. Semantic validation during the internal and external review rounds was performed by using pull requests to 2 different Git branches. After the final approval, profiles and value sets were merged into the main branch of the respective extension module’s repository, which served as the publication branch of that module. Since then, maintenance requests and updates of the extension modules have been handled via GitHub issues. All kinds of relevant changes have become subjects of the internal review, as defined above; major changes (eg, nontechnical corrections) are additionally exposed to the external review.

Implementation guides were created for all 3 extension modules, using the FHIR IG Publisher tool and a customized template for the implementation guides’ HTML pages [21]. The implementation guides were published to GitHub pages, where they remain automatically synchronized with the main branch of the respective repository via continuous integration/continuous development workflows.

### Ethics Approval

This study did not involve any human or animal experiments. No permissions were required to access any data used in this study.

## Results

### Data Set Definition Workflow

We developed an interdisciplinary, iterative, expert consensus–based workflow for the initial definition of domain-specific COVID-19 research data sets based on 2 key requirements. The first key requirement for the content of the data sets was that the content definition (ie, selection of data elements) was to be performed under the full responsibility of a group of medical experts to ensure that the selected data elements were truly those that are required for research in the respective medical specialty. The second key requirement was to produce FAIR (Findable, Accessible, Interoperable, Reusable) digital assets [22], that is, the data set definitions should be represented in FHIR profiles and implementation guides, and these should be registered on open platforms (ie, findable); they should be retrievable through open, free, standard protocols (ie, accessible); they should use only standard, international medical terminologies, such as SNOMED CT and LOINC (ie, interoperable); and they should be released with rich usage guides and examples (FHIR implementation guide) and under a permissive license (ie, reusable).

To fulfill these requirements, the data set definition workflow consists of the following 6 phases: content definition, mapping, quality assurance, publication, an optional public review, and maintenance (Figure 1). In the content definition phase, a group of medical experts from a particular medical specialty are approached by the medical information specialists and asked to provide a list of the data elements that are the most relevant to patient-related COVID-19 research in the respective medical specialty. How the medical expert group compiles the list in detail is left to their discretion (eg, based on systematic literature review or Delphi consensus processes). The medical information specialists only review the provided lists for consistency and redundancy and compile the final content definition in agreement with the medical expert group. In the mapping phase, all data elements are then mapped to international terminologies in consultation with the group of medical experts. Based on these, a logical model and the mappings of data elements to FHIR resources are established. In the quality assurance phase, the FHIR specifications are syntactically validated by using the HL7 FHIR validator as implemented in the FSH Validator Python package (version 0.2.2) [20] and then subjected to a 2-stage review process, during which 2 individual data interoperability and harmonization experts validate the specifications semantically, that is, they validate that the data elements defined by the group of medical experts are appropriately mapped to international standards. After any required changes, the logical model and the FHIR implementation guide are published and are openly accessible to the research community in repositories that fulfill the FAIR criteria as closely as possible, such as ART-DECOR [15] for the logical model and GitHub or the FHIR Implementation Guide registry for the implementation guide [23]. If desired, the initial release of the data set definition can be subjected to public review and balloting processes, which allow stakeholders to provide feedback and suggest changes. The public review and balloting processes provide an opportunity to obtain broader input from and facilitate consensus building among the research community and stakeholders. Any changes resulting from the review and balloting processes can then be incorporated into the data set definition according to the herein presented workflow, and the updated version is released and maintained according to the same workflow. In the maintenance phase, the medical information specialists invite implementers and users of the data set definitions to report any issues they encounter with the definitions via GitHub issues or email, in order to ensure their accuracy and relevance over time. During the maintenance phase, requests for changes or updates to the data set definition should generally be limited to minor issues or corrections, as adding new data elements or making significant modifications to the definition would require running the entire workflow from the beginning.

### Data Set Contents

#### Groups of Medical Experts

In the context of the NAPKON project of the German COVID-19 Research Network of University Medicine [24], so-called *subject- and organ-specific working groups* were established by the voluntary association of medical experts from different medical specialties. In preparation for the domain-specific data set definitions that extend the GECCO core data set, the working groups for infectious diseases (with a focus on immunization), pediatrics, and cardiology were invited by the data set development group to provide up to 50 data elements (with up to 10 response items each) that were of particular interest to their field, concerned patient-related COVID-19 research, and were not already included in the GECCO core data set. For the immunization data set definition, physicians from the COVIM (Collaborative Immunity Platform of the Netzwerk Universitätsmedizin) study for the determination and use of SARS-CoV-2 immunity [25-27] assumed the role of the organ-specific working group, as no such working group had been established previously.

#### Overview

We extended the GECCO core data set by developing domain-specific data set definitions for a total of 250 new data items—48 for the immunization extension module, 150 for the pediatrics extension module, and 52 for the cardiology extension module. These data items were collected, via an iterative consensus-based approach, from the subject- and organ-specific working groups, and they fall under 10 of the 13 data categories of the GECCO data set (Table 1). Data elements and the number of items for each individual extension module are shown in Tables 2, 3, and 4. The full lists of items are shown in the Tables S1-S3 in Multimedia Appendix 1.

**Table 1.** Number of data items per GECCO^a^ data set category for each extension module.

GECCO data category	GECCO extension module		
	Immunization data items (N=48), n	Pediatrics data items (N=150), n	Cardiology data items (N=52), n
Anamnesis and risk factors	13	21	6
Complications	24	47	7
Demographics	—^b^	6	—
Epidemiological factors	—	—	—
Imaging	—	2	36
Laboratory values	1	27	2
Medication	1	35	1
Onset of illness and admission	6	2	—
Outcome at discharge	—	—	—
Study enrollment and inclusion criteria	—	—	—
Symptoms	—	9	—
Therapy	2	1	—
Vital signs	1	—	—

a GECCO: German Corona Consensus.

b Not available.

**Table 2.** Types of data elements in the immunization extension module extending the GECCO^a^ core data set. Shown are the data elements and the FHIR^b^ resource they have been mapped to, as well as the number of items for each data element (ie, different response options).

Category and data element		FHIR resource	Items (N=48), n
**Anamnesis**			
	Chemotherapy	Procedure	1
	Immunosuppressive therapy	Procedure	1
	Regular alcohol intake	Observation	2
**COVID-19 infection and treatment**			
	Disease course	Encounter, Procedure	5
	SARS-CoV-2 infection	Condition	1
	SARS-CoV-2 variant	Observation	1
**Immunization**			
	Contraindications to immunization	Immunization	2
	Immunizations performed	Immunization	3
	Reason for immunization	Immunization	5
	Willingness to receive additional immunization doses	Observation	1
**Immunization reactions**			
	Analgesic or antipyretic drug intake	MedicationStatement	1
	Body temperature	Observation	1
	Complications after immunization	Observation	5
	Medical treatment for adverse reactions	Encounter	3
	Symptoms after vaccination	Condition	16

a GECCO: German Corona Consensus.

b FHIR: Fast Healthcare Interoperability Resources.

**Table 3.** Types of data elements in the pediatrics extension module extending the GECCO^a^ core data set. Shown are the data elements and the FHIR^b^ resource they have been mapped to, as well as the number of items for each data element (ie, different response options).

Category and data element		FHIR resource	Items (N=150), n
**Complications**			
	Complications to COVID-19	Condition	47
**Demographics**			
	Body measures	Observation	6
**Imaging**			
	Echocardiography	Procedure, ImagingStudy	1
	PET-CT^c^	Procedure, ImagingStudy	1
**Immunization**			
	Immunizations performed	Immunization	2
**Laboratory values**			
	Laboratory values	Observation	27
**Medical history**			
	Chronic hematologic diseases	Condition	8
	Chronic kidney diseases	Condition	2
	Congenital disease	Condition	1
	Gastrointestinal diseases	Condition	6
	Medical history stem cells transplant	Condition	2
**Medication**			
	Medication	MedicationStatement, List	35
**Symptoms**			
	COVID-19 symptoms	Condition	9
**Therapy**			
	Hospitalization	Observation	2
	Thoracic drainage	Procedure	1

a GECCO: German Corona Consensus.

b FHIR: Fast Healthcare Interoperability Resources.

c PET-CT: positron emission tomography–computed tomography.

**Table 4.** Types of data elements in the cardiology extension module extending the GECCO^a^ core data set. Shown are the data elements and the FHIR^b^ resource they have been mapped to, as well as the number of items for each data element (ie, different response options).

Category and data element		FHIR resource	Items (N=52), n
**Anamnesis**			
	Chronic cardiologic diseases	Condition	6
**COVID-19−related complications**			
	Cardiologic complications of COVID-19	Condition	7
**Echocardiography**			
	Echocardiography findings	Observation	20
	Echocardiography procedure	Procedure	3
**Electrocardiography**			
	Electrocardiography findings	Observation	11
	Electrocardiography procedure	Procedure	2
**Laboratory values**			
	Laboratory values	Observation	2
**Medication**			
	Angiotensin receptor antagonist	MedicationStatement	1

a GECCO: German Corona Consensus.

b FHIR: Fast Healthcare Interoperability Resources.

All data items were mapped to the appropriate FHIR resources (Observation, Condition, Procedure, MedicationStatement, Encounter, Questionnaire, QuestionnaireResponse, Immunization, ImagingStudy, List, and Specimen), and 26, 14, and 18 profiles (25, 17, and 12 value sets) were created for the immunization, pediatrics, and cardiology extension modules, respectively. The data items that were already part of the GECCO data set and not removed during the data selection step were taken from the GECCO data set and referenced as such in the implementation guides.

The implementation guides for the three extension modules have been published on GitHub pages [28-30]. The source FSH files have been published on GitHub [31-33]. Logical models and data set descriptions are hosted on ART-DECOR, an open collaboration platform for modeling data set definitions, their descriptions, and their terminology bindings [34-36].

## Discussion

### Principal Findings

We herein present an interdisciplinary, iterative, consensus-based workflow for the definition of research data sets, focusing on creating data sets with the most relevant data elements for a particular field of study and on creating universally usable data sets according to the FAIR principles [22]. We applied the workflow to develop 3 GECCO extension modules that contain data items that are relevant for COVID-19–related patient research on infectious diseases (with a focus on immunization), pediatrics, and cardiology. These extension modules complement the GECCO core data set for domain-specified research. The data items are represented in HL7 FHIR profiles and use international terminologies to ensure a harmonized, standardized, and interoperable data set definition for these medical domains. The provision of data according to the extension modules introduced in this paper will enable cross-institutional and cross-country data collection and collaborative research with a particular focus on immunization, pediatrics, and cardiology.

We have specified and implemented an interdisciplinary, iterative, consensus-based workflow for the selection of data items and the development of the data set definition. Close collaboration and constant feedback loops with domain experts from various medical specialties right from the beginning of a project, as performed in this study, are key for the successful development of a useful data set definition. Indeed, since the selection of relevant data items in this study was driven by the end users of the data set, who are the researchers that later will be using the data for their specialized areas of research, the semantic usability of the data sets is guaranteed. Likewise, having medical information specialists develop the formal data set specification ensures the technical interoperability and usability of the data set definition. In this study, we focused on the initial development of interoperable data set definitions for COVID-19–related patient research on infectious diseases (with a focus on immunization), pediatrics, and cardiology. To ensure the continued accuracy and relevance of the data set definitions, such data set definitions should be regularly subjected to public review and balloting processes following the initial development. For example, a revised version of the GECCO data set will undergo HL7 balloting, pending stakeholders’ approval.

Although general interoperability in health care and clinical research is difficult to achieve, we focused on achieving syntactic and semantic interoperability of the data set definitions, which are 2 of the 4 levels into which interoperability can be distinguished, alongside technical and organizational interoperability [8]. We pursued semantic interoperability by using international standardized vocabularies, such as those provided by the LOINC and SNOMED CT vocabularies, to ensure that the meanings of the data elements and their interpretations were unambiguous. We pursued syntactic interoperability by using an open standard for data representation, namely the HL7 FHIR standard, which provides a flexible and extensible framework for exchanging data elements and resources between different systems and applications. We did not focus on organizational interoperability in our work, as this requires coordination and alignment between different health care organizations and stakeholders, which can be challenging in practice. Although we did not specifically address organizational interoperability in our study, we believe that our approach to achieving semantic and syntactic interoperability can contribute to broader efforts toward achieving organizational interoperability over time.

In addition to the successful development of data set definitions, several factors determine a successful deployment or the use of the developed extension modules [37]. First and most importantly, clear and concise documentation of how to implement and provide data using the data set definition is required. For FHIR-based data set definitions, so-called *implementation guides* are used to provide a narrative overview as well as technical details on the data set definition [38]. Thus, we have created and published implementation guides for each of the here developed extension modules. Second, the example implementations of the extension modules serve as a blueprint for developers and data engineers who implement the extension modules for their clinical databases. From our experience with the implementation of the GECCO data set, well-defined example data items may be of equal if not higher importance than the technical description of the data set specification, as developers and engineers tend to use the examples as blueprints for their implementation. Thus, we equipped every FHIR profile defined in the extension modules with at least one example. These examples are incorporated and issued within the implementation guides of the modules. Specifically, we aimed to provide 1 example for each different category of response option per profile. Third, the actual implementation of the extension modules should be part of follow-up infrastructure projects to supply funding and resources for filling the data set definition with actual data. For the GECCO data set, this is ensured by follow-up projects of the German COVID-19 Research Network of University Medicine (“Netzwerk Universitätsmedizin”), such as CODEX+ (Collaborative Data Exchange and Usage), which includes several implementation tasks that are actively using the GECCO data set items [39] and further projects [40-43]. Fourth, once the data set definitions are implemented and leveraged in use cases, additional demands to the data set are likely raised, or issues with existing definitions are revealed. The maintenance of existing definitions (eg, performing technical corrections, evolving the definitions, or adding new items) is, therefore, necessary and must be organized and funded. Last, successful use of the extension modules is also highly dependent on the degree of interoperability of the data set definitions [1,44,45]. For example, the use of questionnaires to assess certain features is common in clinical research. However, depending on the exact wording of the question and the number and wording of response options, results from different studies might not be directly comparable even if they assessed the same features, as the questions and response options differ between studies. In the presented extension modules, several items were at first specified in a questionnaire-like fashion, and the direct implementation of these as Questionnaire resources in FHIR would have limited the applicability of such data elements, especially when aiming to map these elements from an electronic health record system. In these cases, we revised the data element specification to use interoperable concepts rather than questions. Here, repeated consultation with and final approval of the group of medical experts were key to being able to convert questions into interoperable concepts that convey the same information as that intended by the content definition of the group of medical experts. In general, we recommend not to use Questionnaire and QuestionnaireResponse FHIR profiles in cases where the information to be represented can be modeled by using more general, interoperable concepts and FHIR resources.

The challenges of creating and harmonizing COVID-19 data sets are not unique to our work, and although initiatives, such as the Clinical Data Interchange Standards Consortium (CDISC), have released guidance on how to represent COVID-19 research data in a standardized format [46], the actual selection of the relevant biomedical concepts to be represented is left to the implementers. We explicitly selected the data elements for COVID-19–related patient research that are the most relevant for further characterizing patients with respect to research in infectious diseases (with a focus on immunization), pediatrics, and cardiology. However, we recognize the need for ongoing collaboration and standardization efforts to maximize interoperability and facilitate data sharing and analysis. Such efforts include integrating the GECCO data set with other COVID-19–related data sets and standards, both within and between countries. For example, we are currently harmonizing the GECCO data set with the ORCHESTRA (Connecting European Cohorts to Increase Common and Effective Response to SARS-CoV-2 Pandemic) project, which intends to create a harmonized and standardized data set for a pan-European cohort for COVID-19 research [40]. To facilitate the mapping of the data items that were developed in our work and represented in HL7 FHIR to the CDISC Study Data Tabulation Model standard, the organizations behind the two standards have collaboratively developed a comprehensive implementation guide, thereby enabling mapping between the different standards, ensuring compatibility, and facilitating interoperability across systems [47]. Moving forward, we encourage developers of tools and resources to facilitate the mapping and harmonization of different data standards, and we look forward to continued collaboration with the wider research community to address these challenges and advance COVID-19 research.

### Conclusion

We herein introduce the development workflow and the resulting data set definitions for GECCO extension modules for the immunization, pediatrics, and cardiology domains. We have defined and implemented a workflow in which interdisciplinary teams of medical domain experts, medical information specialists, and FHIR developers closely collaborate in an iterative, consensus-based fashion for the successful development of useful and interoperable data set definitions. This workflow may serve as a blueprint for further data set definition projects, such as the further development of data set definitions for extending the GECCO core data set. The extension modules described in this work have been validated and published. Their implementation and active use are anticipated in the context of current nationwide COVID-19 research networks in Germany.

## Supplementary material

10.2196/45496 Multimedia Appendix 1 Supplementary tables.

## Abbreviations

CDISC: Clinical Data Interchange Standards Consortium
CODEX+: Collaborative Data Exchange and Usage
COVIM: Collaborative Immunity Platform of the Netzwerk Universitätsmedizin
FAIR: Findable, Accessible, Interoperable, Reusable
FHIR: Fast Healthcare Interoperability Resources
FSH: Fast Healthcare Interoperability Resources Shorthand
GECCO: German Corona Consensus
HL7: Health Level Seven International
LOINC: Logical Observation Identifiers Names and Codes
NAPKON: Nationales Pandemie Kohorten Netz
ORCHESTRA: Connecting European Cohorts to Increase Common and Effective Response to SARS-CoV-2 Pandemic
SNOMED CT: Systematized Nomenclature of Medicine-Clinical Terms
