# Supplementary material for: Interoperable, Domain-Specific Extensions for the German Corona Consensus (GECCO) COVID-19 Research Data Set Using an Interdisciplinary, Consensus-Based Workflow: Data Set Development Study
Source: JMIR Med Inform. 2023 Jul 18;11:e45496. doi: 10.2196/45496 (PMC10368099; doi:10.2196/45496)
Supplement: Multimedia Appendix 1 [file medinform-v11-e45496-s001.pdf]

Supplementary Appendix

The following tables show the data items that are included in the immunization extension module (Table S 1), the pediatrics extension module (Table S 2), and the cardiology extension module (Table S 3). Note that these tables list only the data items of the extension modules that are not included in the GECCO core dataset and that the complete dataset definition for each module consists of the GECCO core dataset together with the data items of the extension module.

GECCO Immunization extension module

Category	Data Element	FHIR Resource	Item
**Anamnesis**	Chemotherapy	Procedure	Chemotherapy
	Immunosuppressive therapy	Procedure	Immunosuppressive therapy
	Regular Alcohol Intake	Observation	Frequency
			Quantity
**COVID-19 infection & treatment**	Disease course	Encounter, Procedure	No symptoms
			Treated at home
			Treated at hospital with oxygen therapy
			Treated at hospital without oxygen therapy
			Treated at intensive care unit
	SARS-CoV-2 infection	Condition	SARS-CoV-2 infection
	SARS-CoV-2 variant	Observation	SARS-CoV-2 variant
**Immunization**	Contraindications to immunization	Immunization	Allergy
			Pregnancy
	Immunizations performed	Immunization	Date
			Lot number
			Type
	Reason for immunization	Immunization	Everybody gets vaccinated
			Protection of private environment from infection/disease
			Protection of themself from infection/disease
			Protection of work environment from infection/disease
			Worrying about disadvantages
	Willingness to receive additional immunization doses	Observation	Willingness to receive additional immunization doses
**Immunization reactions**	Analgesic or antipyretic drug intake	MedicationStatement	Analgesic or antipyretic drug intake
	Body temperature	Observation	Body temperature after vaccination
	Complications after immunization	Observation	Allergic reaction after immunization
			Injection site erythema
			Injection site pain at rest
			Injection site pain during pressure/movement
			Injection site swelling
	Medical treatment for adverse reactions	Encounter	Ambulatory
			Inpatient
			No treatment
	Symptoms after Vaccination	Condition	Chill

			Diarrhea
			Difficulty breathing
			Dyspnea
			Exhaustion
			Fatigue
			Feeling feverish
			Fever
			Fever with chills
			Joint pain
			Liquid stool
			Loose stool
			Muscle pain
			Nausea
			Soft stool
			Vomiting symptom

Table S 1 Data items in the immunization extension module extending the GECCO core dataset. Shown are the data elements and the FHIR resource they have been mapped to, as well as the items for each data element (i.e., different response options).

GECCO Pediatrics extension module

Category	Data Element	FHIR Resource	Item
**Complications**	Complications to COVID-19	Condition	Anemia
			Arterial aneurysm
			Ascites
			Aspergillosis
			Bacterial arthritis
			Bacterial endocarditis
			Bacterial meningitis
			Bacterial osteomyelitis
			Bronchiolitis
			Bronchitis
			Chlamydial infection
			Chronic fatigue syndrome
			Colitis
			Disease caused by Adenovirus
			Disease caused by Coronaviridae
			Disease caused by Human bocavirus
			Disease caused by Rhinovirus
			Disorder of liver
			Haemophilus influenzae infection
			Human metapneumovirus infection
			Ileitis

			Infection caused by Candida albicans
			Infection caused by Escherichia coli
			Infection caused by Klebsiella
			Infection caused by Pseudomonas aeruginosa
			Infection caused by Staphylococcus aureus
			Infection caused by Streptococcus viridans group
			Infection caused by enterococcus
			Infection of bloodstream
			Influenza
			Invasive Group A beta-hemolytic streptococcal disease
			Invasive Streptococcus pneumoniae disease
			Legionella infection
			Meningococcal infectious disease
			Mycoplasma infection
			Organic mental disorder
			Parainfluenza
			Peritonitis
			Pertussis
			Post-acute COVID-19
			Procedure needed Where Associated procedure = Resuscitation
			Pyelonephritis
			Respiratory syncytial virus infection
			Seizure disorder
			Streptococcus agalactiae infection
			Streptococcus pyogenes infection
			Syncope
**Demographics**	Body measures	Observation	Birth height
			Birth height (percentile)
			Body mass index
			Body mass index (percentile)
			Head circumference
			Head circumference (percentile)
**Imaging**	Echocardiography	Procedure, Imaging Study	Echocardiography
	PET-CT	Procedure, Imaging Study	Positron emission tomography with computed tomography
**Immunization**	Immunizations performed	Immunization	Viral vector vaccine
			mRNA vaccine
**Laboratory values**	Laboratory values	Observation	Alanine Aminotransferase
			Albumin
			Amylase
			Calprotectin
			Cells in CSF
			Complement C3
			Complement C4
			Creatine kinase
			Creatine kinase.MB

			Erythrocyte sedimentation rate
			Glucose in CSF
			Hematocrit
			IgG
			Interleukin 10
			Interleukin 2 Receptor Soluble
			Lactate in CSF
			Lipase
			Natural killer cell function
			Neutrophil cytoplasmic Ab
			Nuclear Ab
			Protein in CSF
			Prothrombin time (PT)
			SARS-CoV-2 RT in stool
			SARS-CoV-2 RT in urine
			Sodium
			Triglyceride
			Urea
**Medical history**	Chronic Hematologic Diseases	Condition	Blood coagulation disorder
			Glucose-6-phosphate dehydrogenase deficiency anemia
			Hemolytic anemia
			Iron deficiency anemia
			Myelodysplastic syndrome
			Neutropenic disorder where Clinical course = Chronic
			Sickle cell-hemoglobin SS disease
			Thalassemia
	Chronic Kidney Diseases	Condition	Disorder of the urinary system where Occurrence = Congenital
			Kidney disease where Occurrence = Congenital
	Congenital Disease	Condition	Congenital Disease
	Gastrointestinal Diseases	Condition	Allergy to cow's milk protein
			Celiac disease
			Chronic constipation
			Disorder of bile duct
			Disorder of gastrointestinal tract
			Inflammatory bowel disease
	Medical History Stem Cells Transplant	Condition	History of bone marrow transplant
			History of peripheral stem cell transplant
**Medication**	Medication	MedicationStatement	Blood product
			Bronchodilator
			Inotropic agent
			Medicinal product acting as antibacterial agent
			Medicinal product acting as hemostatic
			Product containing anakinra
			Product containing aspirin
			Product containing human immunoglobulin
			Product containing nitric oxide

			Product containing tocilizumab
			Vasopressor
		MedicationStatement, List	Alkylating agent
			Anti-CD20 antibody
			Anti-CD3 antibody
			Anti-CD3/CD19 antibody
			Anti-CD52 antibody
			Antimetabolite
			B cell activating factor inhibitor
			Calcineurin inhibitor
			Cytotoxic T-lymphocyte-associated protein 4 inhibitor
			Equine antithymocyte immunoglobulin
			Immunoglobulin E
			Integrin inhibitor
			Interferon
			Interleukin 1 receptor antagonist
			Interleukin 17 receptor antagonist
			Interleukin 2 receptor antagonist
			Interleukin 6 receptor antagonist
			Janus kinase inhibitor
			Mammalian target of rapamycin-Kinase inhibitor
			Protein-tyrosine kinase inhibitor
			Sphingosine analogue
			Steroid
			Tumor necrosis factor alpha inhibitor
			interleukin 23 receptor antagonist
**Symptoms**	COVID-19 Symptoms	Condition	Delirium
			Eruption of skin
			Large liver
			Myoclonus
			Pain in throat
			Palmar erythema
			Raspberry tongue
			Splenomegaly
			Swallowing painful
**Therapy**	Hospitalization	Observation	Intensive care treatment duration
			Total length of stay
	Thoracic Drainage	Procedure	Thoracic Drainage

Table S 2 Data items in the pediatrics extension module extending the GECCO core dataset. Shown are the data elements and the FHIR resource they have been mapped to, as well as the items for each data element (i.e., different response options).

GECCO Cardiology extension module

Category	Data Element	FHIR Resource	Item
**Anamnesis**	Chronic cardiologic diseases	Condition	Atrial fibrillation
			Atrial flutter
			Cardiomyopathy
			Congenital heart disease
			Heart failure
			History of coronary artery bypass grafting
**COVID-19-related complications**	Cardiologic complications of COVID-19	Condition	Bacterial respiratory infection
			Cardiogenic shock
			Complete atrioventricular block
			Myocarditis
			Pericardial effusion
			Ventricular fibrillation
			Viral disease
**Echocardiography**	Echocardiography findings	Observation	Abscess of heart
			Aortic valve regurgitation
			Aortic valve stenosis
			Heart valve disorder
			Left Ventricular Ejection Fraction
			Left ventricular hypertrophy
			Left ventricular wall motion abnormality
			Mitral valve regurgitation
			Mitral valve stenosis
			Paradoxical cardiac wall motion
			Pericardial effusion
			Pulmonic valve regurgitation
			Pulmonic valve stenosis
			Right ventricular hypertrophy
			Thrombosis
			Tricuspid annular plane systolic excursion (TAPSE)
			Tricuspid valve regurgitation
			Tricuspid valve stenosis
			Vegetation of heart
			Ventricular hypertrophy
	Echocardiography procedure	Procedure	Date
			Echocardiography
			Type of echocardiography
**Electrocardiography**	Electrocardiography findings	Observation	Atrial ectopics
			Atrioventricular Block
			Bundle Branch Block
			Inverted T wave
			Low QRS voltages
			Premature ventricular contractions
			QRS Axis

			QRS Interval
			QT Interval
			ST Interval
			Sinus rhythm
	Electrocardiography procedure	Procedure	12 lead electrocardiogram
			Date
**Laboratory Values**	Laboratory values	Observation	Troponin I
			Troponin T
**Medication**	Angiotensin receptor antagonist	MedicationStatement	Angiotensin Receptor Antagonist

Table S 3 Data items in the cardiology extension module extending the GECCO core dataset. Shown are the data elements and the FHIR resource they have been mapped to, as well as the items for each data element (i.e., different response options).
